# Supplementary material for: Structural-Activity Relationship of Rare Ginsenosides from Red Ginseng in the Treatment of Alzheimer’s Disease
Source: Int J Mol Sci. 2023 May 11;24(10):8625. doi: 10.3390/ijms24108625 (PMC10218514; doi:10.3390/ijms24108625)
Supplement: Supplementary file 1 [file ijms-24-08625-s001.zip › ijms-2389425-supplementary.pdf]

Supplementary Information

Structure-activity relationship of rare ginsenosides in red ginseng and their mechanism of prevention and treatment of Alzheimer's disease

Xianwen Ye [1,2,3], Haixia Zhang [1,2], Qian Li [1,2], Hongmin Ren [1,2], Xinfang Xu [1,2,3,*] and Xiangri Li [1,2,3,*]

1 Centre of TCM Processing Research, Beijing University of Chinese Medicine, Beijing 102488, China; 20210941399@bucm.edu.cn (X.Y.); 20220935103@bucm.edu.cn (H.Z.); 20220935102@bucm.edu.cn (Q.L.); renhongmin0607@bucm.edu.cn (H.R.)

2 Beijing Key Laboratory for Quality Evaluation of Chinese Materia Medica, Beijing University of Chinese Medi-cine, Beijing 102488, China

3 Institute of Regulatory Science for Traditional Chinese Medicine, Beijing University of Chinese Medicine, Beijing 102488, China

* Correspondence: lixiangri@sina.com (X.L.); 202102008@bucm.edu.cn (X.X.)

Table S1. Docking results and hydrogen bond related information between ginsenosides and inflammation factors

	PDB ID	Ligand	Libscore	Affinity kcal/mol	Ligand atom	Amino acid residue atom	Bond length (A)	Bond angle (°)
iNOS	4NOS	*R*-Rh1	85.667	-10.0	O26	LYS445:HZ1	2.54	134.41, 96.96
		S-Rh1	87.771	-10.2	O30	LYS405:HZ3	2.09	153.08, 102.20
					H103	HIS424:O	2.81	90.50, 138.43
					H107	ILE423:O	2.43	126.92, 101.07
		R-Rg3	93.512	-8.9	H84	HIS404:O	2.03	174.09, 162.85
					H85	HIS404:O	2.12	123.82, 121.01
		S-Rg3	118.709	-8.4	H84	ILE433:O	2.23	107.17, 156.89
					H88	ILE433:O	2.39	97.12, 117.52
					H107	HIS424:O	2.23	95.69, 141.62
IL-6	1ALU	*R*-Rh1	101.241	-6.0	H104	THR43:OG1	1.52	142.82, 116.72
					H107	ARG104:O	2.24	112.77, 119.02
		S-Rh1	109.137	-5.3	H102	GLN156:OE1	1.86	149.61, 112.09
					H103	GLN159:OE1	2.22	115.99, 90.68
					H104	GLN152:O	2.52	157.29, 169.59
					H104	GLN159:OE1	2.77	128.45, 156.72
					H107	ARG104:O	2.58	121.43, 111.14
		R-Rg3	119.677	-6.0	O45	SER107:HN	2.93	131.03, 124.76
					H100	ARG104:O	2.35	94.33, 120.17
					H108	ARG104:O	2.92	136.68, 103.42
					H112	ASP160:OD1	2.04	104.35, 136.91
		S-Rg3	131.397	-5.4	O22	GLN159:HE22	2.76	118.65, 99.81
					H84	GLN159:OE1	2.47	108.67, 114.54
					H88	GLN152:O	1.80	147.98, 156.12
					H97	THR43:O	2.91	132.60, 101.60
TNF-α	2AZ5	*R*-Rh1	95.795	-8.4	H103	LEU157:OXT	2.92	95.16, 142.23
		S-Rh1	96.283	-8.4	H79	LEU157:OXT	2.51	139.81, 146.59
					H81	LEU157:OXT	1.95	97.83, 143.40
		R-Rg3	140.434	-8.5	O45	ASN39:HD22	2.44	121.67, 104.61
					O47	ASN39:HD22	2.69	153.00, 107.39
					H97	PRO12:O	2.30	125.02, 109.72
					H101	ALA156:O	3.03	91.94, 138.03

S-Rg3	152.796	-8.1	H97	PRO12:O	2.55	115.00, 97.94
			H97	LYS11:O	2.17	152.38, 95.96
			H100	LYS11:O	2.07	118.19, 149.27
			H101	ALA156:O	2.59	95.56, 138.64
			H112	HIS15:NE2	2.84	106.22, 102.10

Table S2. Docking results and hydrogen bond related information between ginsenosides and target protein of AD

	PDB ID	Ligand	Libscore	Affinity kcal/mol	Ligand atom	Amino acid residue atom	Bond length (A)	Bond angle (°)
NLRP3	2NAQ	Rk1	127.969	-7.1	H124	ALA67:O	2.45	145.85, 107.36
		Rg5	118.046	-6.3	H89	GLU63:OE1	2.23	119.35, 106.80
					H73	GLU89:OE2	2.71	92.52, 141.77
		Rk3	102.623	-7.0	O31	LEU39:HN	1.91	142.69, 94.50
					H101	LEU27:O	2.51	94.68, 93.69
					H104	TYR30:O	2.52	150.35, 121.69
		Rh4	125.641	-7.1	H67	LEU39:O	2.53	92.54, 142.42
ASC	6KI0	Rk1	132.283	-9.6	O27	ARG354:HH22	2.81	107.08, 103.60
					O38	ARG354:HH22	2.18	110.82, 98.99
					O40	ALA427:HN	2.91	119.48, 104.08
					H81	ASP207:OD1	1.64	156.26, 134.48
					H84	ASP207:OD1	2.29	132.19, 167.13
					H95	SER423:O	2.63	127.11, 142.98
		Rg5	128.829	-8.7	H89	GLY397:O	2.59	131.47,

								108.53
		Rk3	118.049	-10.9	H101	SER423:O	2.1	171.07,
								127.99
		Rh4	114.324	-11.4	O39	ARG354:HH12	3.03	94.32,
					O41	ALA427:HN	2.81	118.86
					H101	SER423:O	1.88	129.84,
								106.48
								141.96,
								154.29
Caspase-1	5MTK	Rk1	121.568	-5.8	O40	HIS237:HD1	2.21	138.02,
					H95	GLY238:O	1.80	119.24
								143.47,
								119.98
		Rg5	120.325	-6.5	O38	HIS237:HD1	1.84	166.66,
					H88	GLY238:O	1.80	104.83
					H107	GLY287:O	2.52	127.32,
								125.33
								135.79,
								137.09
		Rk3	121.852	-6.0	H101	ASP381:O	2.31	95.54,
					H101	ARG383:O	1.91	138.28
								132.23,
								129.73
		Rh4	100.136	-6.7	H66	ASP288:OD1	2.74	134.80,
					H100	ARG178:O	2.34	151.40
								100.97,
								153.33

Metabolomics study of urine

QC sample preparation

Take the same amount of urine from each mouse in the normal control group, model group, and drug administration group, mix it well, and prepare quality control samples according to the above procedure.

The chromatographic conditions

Mobile phase: acetonitrile-0.1% formic acid (containing 10 mM ammonium acetate); Column temperature: 25°C; Flow rate: 0.6 mL/min; Column: Waters ACQUITY UPLC BEH C18 (2.1*100mm, 1.7μm); Injection volume: 1μL, scanning range: 100-1500. As shown in **Table S3.**

Table S3. Gradient elution conditions of urine samples

Time（min）	acetonitrile（%）	0.1% formic acid solution (containing 10mM ammonium acetate)
0	2	98
15	95	5
18	95	5
18.1	2	98
23	2	98

Mass spectrometry conditions

Matrix-assisted laser desorption ionization (MALDI): positive ion mode; Capillary voltage: 3kV, cone hole voltage: 40V; Desolvent temperature: 450°C; Ion source temperature: 120°C; Desolvent gas flow rate: 700L/h; Taper hole gas flow rate: 50L/h; The collection mass ranges from 100 AMU to 1500 AMU. Collision energy: low energy 4eV, high energy 15eV; Data acquisition mode: Centroid; Scan frequency: 30s; TOF operation mode: V mode. Reference solution: Leucine enkephalin (LE). Liquid data acquisition software MassLynx4.1.

Negative ion mode; Capillary voltage: 2kV, cone hole voltage: 40V; Desolvent temperature: 400°C; Ion source temperature: 120°C; Desolvent gas flow rate: 800L/h; Taper hole gas flow rate: 50L/h; The collection mass ranges from 100 AMU to 1500 AMU. Collision energy: low energy 4eV, high energy 15eV; Data acquisition mode: Centroid; Scan frequency: 30s; TOF operation mode: V mode. Reference solution:

Leucine enkephalin (LE). Liquid data acquisition software MassLynx4.1.

Methodological investigation

The instrument precision, method reproducibility, and system stability of the UPLC-MS method were investigated by the relative standard deviation (RSD) between the retention time (tR) of mass/charge ratio (m/z) and the ion peak intensity in QC samples.

Precision inspection of the instrument: the same QC sample was injected and analyzed three times in a row, and three identical m/z were selected from each analysis result to calculate the RSD value of tR and its peak intensity respectively, and the RSD value was required to be less than 10%.

Methods Reproducibility investigation: Three QC samples were prepared in parallel and analyzed by injection. Three identical m/z were selected from each analysis result, and the RSD values of tR and its peak intensity were calculated respectively, and the RSD values were required to be less than 15%.

System stability inspection: In the process of sample data collection, QC samples should be tested once for every 10 samples tested. After sample testing is completed, three QC results in the process are taken, three identical m/z are selected, and the RSD values of tR and its peak intensity are calculated respectively, and RSD values are required to be less than 20%.

Liquid data processing

Data processing is mainly carried out by the Masslyx workstation, where the MarkerLynxXS module can perform multivariate statistical analysis on liquid data. The structure of the composite can be further identified by scanning the ion fragments under the high-energy surface. The collected data of liquid is a 3D data point including retention time, M/Z, and intensity. This data point must be transformed into a

two-dimensional data point for subsequent multivariate statistical analysis. Data transformation can be done by the markerlynxXS module in Masslynx.

Urine metabolomics results

Methodological investigation

Three tR_m/z in positive ion mode (4.89_379, 10.45_302, 11.87_330) and three tR_m/z in negative ion mode (2.54_231, 4.94_377, 5.70_427) of urine QC samples were selected respectively.

The RSD of tR and its ion peak area was calculated respectively to investigate the instrument precision, method reproducibility, and system stability of the UPLC-MS method. The specific results are shown in **Table S4**.

The results show that the precision, stability, and reproducibility of RSD of UPLC-MS retention time in positive ion mode were 0.07~0.09, 0.15~0.27, and 0.05~0.27, respectively, and the RSD values of the corresponding ion peak area were 2.53~9.85, 2.41~9.57, and 0.96~7.70; The precision, stability, and reproducibility of UPLC-MS retention time in negative ion mode were 0.27~1.14, 0.37~1.13 and 0.43~1.25, respectively, and the RSD values of corresponding ion peak area were 2.07~3.43, 0.77~9.39 and 4.08~12.52, respectively. The results show that the method is accurate, reliable, and can meet the requirements of metabonomics research.

Table S4. The results of UPLC-MS methodology investigation in urine metabonomics (*Mean*, *n*=5)

t_R-*m/z*	Precision (RSD,%)		Stability (RSD,%)		Repeatability (RSD,%)	
	t_R	Peak area	t_R	Peak area	t_R	Peak area
**Positive ion mode**						
4.89-379	0.09	2.53	0.27	3.09	0.27	2.44

10.45-302	0.09	7.84	0.19	2.41	0.16	0.96
11.87-330	0.07	9.85	0.15	9.57	0.05	7.70
**Negative ion mode**						
2.54-231	1.14	3.43	0.95	5.47	0.95	4.08
4.94-377	0.38	2.24	0.37	9.39	0.43	12.52
5.70-427	0.27	2.07	1.03	0.77	1.25	1.83

Total ion flow diagram analysis

Liquid chromatography-mass spectrometry (LC-MS) is usually used for the analysis of metabonomic samples, and the full component analysis of complex samples is realized by positive and negative ion models. In this experiment, the total ion flow diagram of the urine sample in positive and negative ion modes is shown in **Figure S1** and **Figure S2**. As can be seen in **Figure S1**, the chemical composition of urine samples in positive ion mode is very complex and the chemical composition of each group of samples is similar, so it is difficult to distinguish from the total ion flow diagram. Compared with the model group, the total ion current diagram of 8-12 min in the control group was different from that in the model group, and it would change obviously after administration.

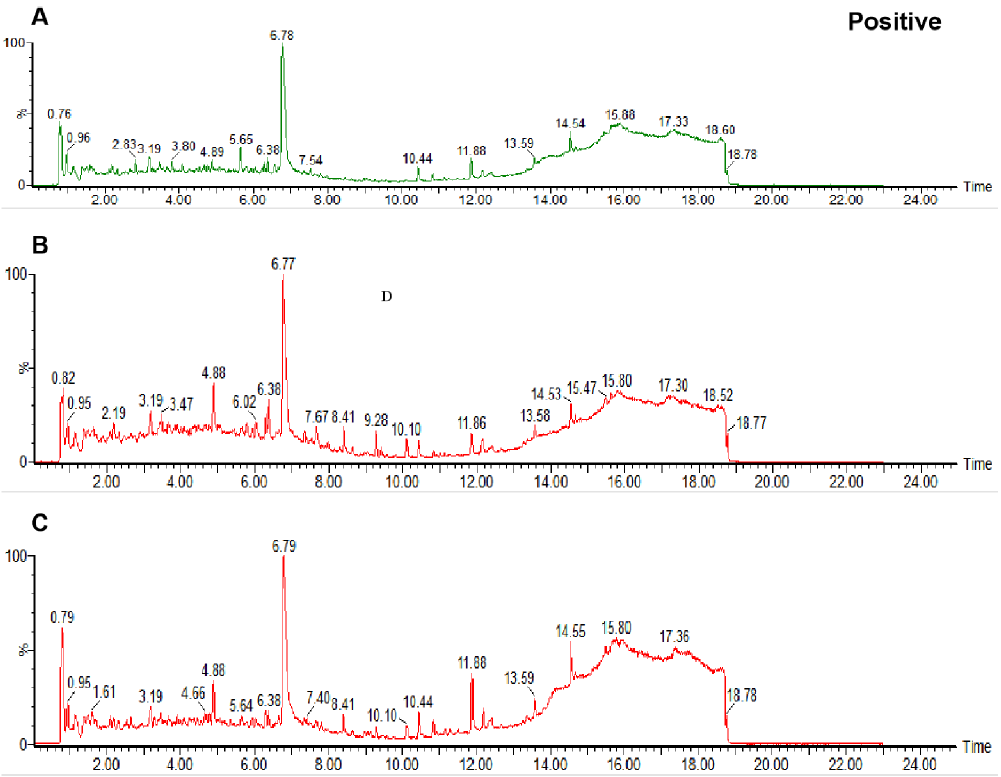

Figure S1 The total ion flow diagram of urine sample in positive ion mode (A: Control; B: Model group; C:Rh4 group)

As can be seen in **Figure S2**, the chemical composition of urine samples in negative ion mode is very complex and the chemical composition of each group of samples is similar, so it is difficult to distinguish from the total ion flow diagram. The metabolites of urine are different in the positive ion mode and the negative ion mode. The sample has a uniform peak in the positive ion mode, and it is mainly a polar metabolite in the negative ion mode.

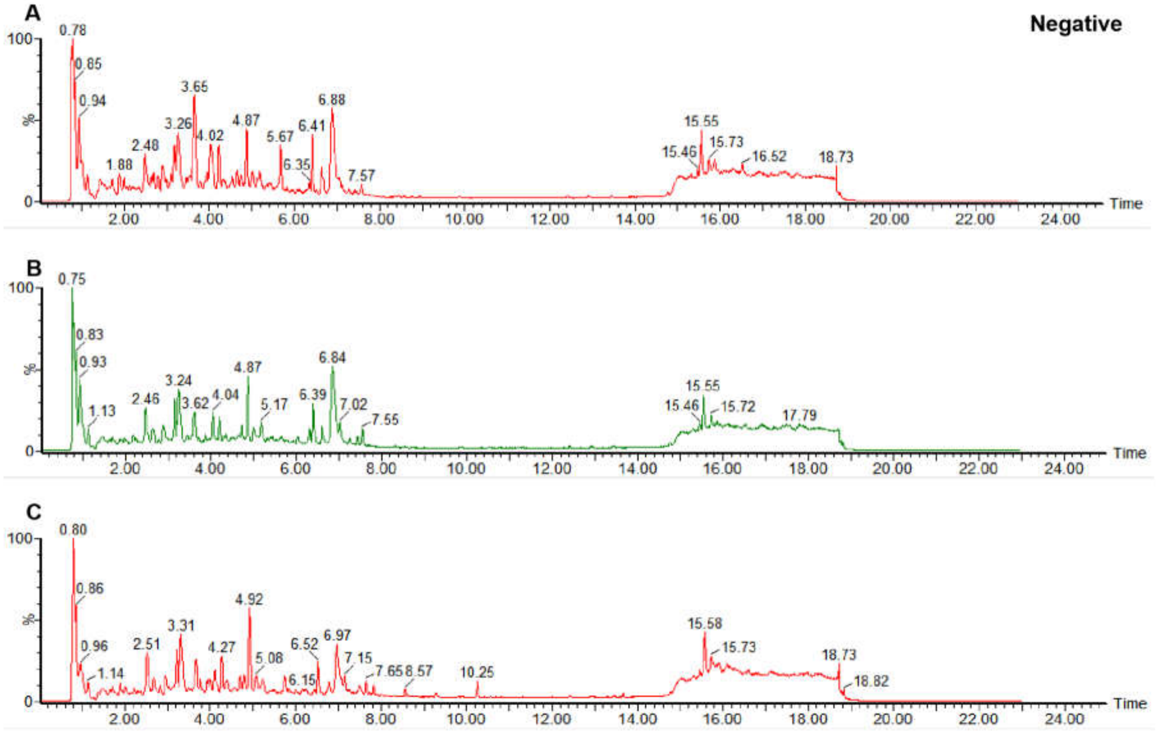

Figure S2 The total ion flow diagram of urine sample in negative ion mode (A: Control; B: Model group; C:Rh4 group)
